# Self medication with antibiotics in Yogyakarta City Indonesia: a cross sectional population-based survey

**DOI:** 10.1186/1756-0500-4-491

**Published:** 2011-11-11

**Authors:** Aris Widayati, Sri Suryawati, Charlotte de Crespigny, Janet E Hiller

**Affiliations:** 1 Faculty of Pharmacy, Sanata Dharma University, Yogyakarta, Indonesia; 2 Faculty of Medicine, Gadjah Mada University, Yogyakarta, Indonesia; 3 School of Nursing, University of Adelaide, Australia; 4 School of Population Health and Clinical Practice, University of Adelaide, Australia; 5 Faculty of Health Sciences, Australian Catholic University, Melbourne, Australia

## Abstract

**Background:**

Self medication with antibiotics has become an important factor driving antibiotic resistance. This study investigated the period prevalence, patterns of use, and socio-demographic factors associated with self medication with antibiotics in Yogyakarta City Indonesia. This cross-sectional population-based survey used a pre-tested questionnaire which was self-administered to randomly selected respondents (over 18 years old) in Yogyakarta City Indonesia in 2010 (N = 625). Descriptive statistics, chi-square and logistic regression were applied.

**Results:**

A total of 559 questionnaires were analyzed (response rate = 90%). The period prevalence of self medication with antibiotics during the month prior to the study was 7.3%. Amoxicillin was the most popular (77%) antibiotic for self medication besides ampicilline, fradiomisin-gramisidin, tetracycline, and ciprofloxacin to treat the following symptoms: the common-cold including cough and sore throat, headache, and other minor symptoms; with the length of use was mostly less than five days. Doctors or pharmacists were the most common source of information about antibiotics for self medication (52%). Antibiotics were usually purchased without prescription in pharmacies (64%) and the cost of the purchases was commonly less than US $1 (30%). Previous experience was reported to be the main reason for using non-prescribed antibiotics (54%). There were no socio-demographic variables significantly associated with the actual practice of using non-prescribed antibiotics. However, gender, health insurance, and marital status were significantly associated with the intent to self medicate with antibiotics (P < 0.05). Being male (Odds Ratio = 1.7 (1.2 - 2.6)) and having no health insurance (Odds Ratio = 1.5 (1.0 -2.3)) is associated with the intent to self medicate with antibiotics.

**Conclusions:**

This study is the first population-based study of self-medication with antibiotics among the Indonesian population. Usage of non-prescribed antibiotics as well as intent of doing so is common across socio-demographic categories. Given the findings, factors influencing people's intentions to self medicate with antibiotics are required to be investigated to better understand such behavior. Impact of health insurance coverage on self medication with antibiotics should also be further investigated.

## Background

Self medication with antibiotics or the use of non-prescribed antibiotics including leftover antibiotics is common in both developed and developing countries, in which the point prevalence ranges from 3% to 75% [1-5]. Non-prescribed antibiotics may be used inappropriately due to a lack of health professional supervision. For example, previous studies in Jordan [6], in Lao People's Democratic Republic [7], and in European countries [8] found that non-prescribed antibiotics mostly were used for treating the common cold, a viral condition for which antibiotics treatment is ineffective [9]. As a consequence, self medication with antibiotics may be associated with undesirable effects, such as decreased effectiveness and worsening clinical conditions and has become an important factor driving antimicrobial resistance [10]. Such consequences potentially jeopardize the health of the individual who self-medicates as well as society as a whole.

Problems related to self medication with antibiotics particularly in the developing world, are complex as they are linked to other issues, such as poverty, lack of access to medicines and information regarding medicines, poor quality of health care facilities, and weak implementation of regulation related to medicines. For example, limited purchasing power is associated with self medication with antibiotics among the population in Kerala India [11], Nigeria [12], the Philippines [13], Latino adults in the United States of America [14], and the non-Arab population in the United Arab Emirates (UAE) [15]. In addition, because of economical constraints, poor people may purchase insufficient amounts of non-prescribed antibiotics [16]. They may be forced to purchase antibiotics without a prescription at a low price in common kiosks (i.e. with lay sellers), which can be poor quality or counterfeit. Other issues related to poor quality and inadequate access to the health care service also exist, such as, waiting times, limited supply of medicines, unacceptable practices of health professionals in health care facilities, the high cost of transportation to reach health centers, and having no health insurance [5,11]. Such issues potentially persuade people to self medicate instead of seeking a medical consultation.

Research understanding patterns of utilization of antibiotics and factors associated with such use is essential for improving the appropriate use of antibiotics particularly in less developed countries. Such epidemiological information is useful in guiding the formulation of educational interventions [17], especially to understand features of antibiotic use, to identify possible causes, and to define goals of future interventions [18].

In Indonesia antibiotics are legally categorised as prescription-only medicines whose sale is restricted to pharmacies [19]. Drug stores are not allowed to sell prescription-only medicines, while kiosks can only sell over- the-counter (OTC) medicines, such as paracetamol and vitamin C [20]. However, antibiotics can be easily purchased without prescription and are accessible from those vendors.

Although information on the prevalence, patterns of utilization of non-prescribed antibiotics and socio-demographic factors has been available in both developed and developing countries, in Indonesia such studies are uncommon. One previous study [5], in 2001 to 2002, reported the patterns of self medication with antibiotics and socio-demographic factors of such use among individuals visiting public healthcare facilities. However, that study was not a population-based survey and therefore was neither representative nor could exclude sample bias. In addition, that study did not explore the reasons of self medication using antibiotics, which are important for understanding such behavior. This present study is the first population-based survey in an urban area in Indonesia to investigate the prevalence, utilization patterns of self medication with antibiotics including the reasons, and socio-demographic factors of such use.

## Methods

### Study area

This study was conducted in Yogyakarta City Indonesia. The city is one of five regencies of Yogyakarta Province and lies in Java Island. Among these regencies the population of Yogyakarta City in 2010 was the smallest (11.2% or 388,088 persons). However, the population density was the highest (about 14,000 persons per kilo meters square) with the area comprising approximately 1% of total area of the province [21]. Although individuals of Javanese descent predominate, the city is known as a multiethnic city given the variety of ethnic groups who live there.

### Study design, sample size calculation, and sampling

This study was a population-based cross-sectional survey. The study population was adults (over 18 years old) living in Yogyakarta City. Respondents were randomly selected using a multi-stage clustered random sampling technique. The required sample size adjusted for cluster was 640 respondents. This was estimated based on the following factors: expected proportion of the population self medicating with antibiotics in developing countries (P) = 50%; tolerated error/margin of error (*d*) = 0.05; confidence interval (*CI*) = 95%; attrition rate = 1/10; design effect = 1.5 [22].

Yogyakarta City consists of 14 districts (*kecamatan*); 45 sub-districts (*kelurahan*); and 2524 neighborhoods (*RT*). One neighborhood consists of about 50 to 70 households. The initial cluster was district, followed by sub-district, and neighborhood. The probability proportionate to size (PPS) was applied to calculate of the clusters in each stage. In the first stage, all fourteen districts of Yogyakarta City were involved. In the second stage, 30% of the total numbers of sub-districts located in each district were randomly selected, resulting in 15 selected sub-districts. In the third stage, 5% of the total neighborhoods located in each sub-district chosen were randomly selected, resulting in 41 selected neighborhoods. Fifteen to sixteen households were randomly selected in every neighborhood, resulting in 640 selected households. One adult person was randomly selected from every household chosen using a systematic grid [23], as shown in Table 1. The process of the selection is as follows: once households had been selected, they were numbered systematically from 1 to 12; a list of all adults living in a particular household visited was made, all males were firstly listed from eldest to youngest and then females in the same way or vice versa; finally, a particular person was selected using the grid, based on the number of the household (between 1 and 12) and the number of eligible persons in the household.

**Table 1:** The grid for selecting individuals in the selected households (Source: Hoinville et al, 1977:82 cited in de Vaus, 2002: 77 [23])

Assigned number of household	Total number of eligible persons					
	1	2	3	4	5	6 or more
1 or 2	1	1	2	2	3	3
3	1	2	3	3	3	5
4 or 5	1	2	3	4	5	6
6	1	1	1	1	2	2
7 or 8	1	1	1	1	1	1
9	1	2	3	4	5	5
10 or 11	1	2	2	3	4	4
12	1	1	1	2	2	2

### Survey instrument and data collection

A questionnaire as an instrument of this survey was developed based on literature in which self medication with antibiotics was examined in across Asian countries [5,24,25]. A combination of open-ended and close-ended was used. Questions about patterns of SMA included the symptoms for which antibiotics were being used; the types of antibiotic; the duration of use; the price paid; the reasons for self medication; the sources of antibiotics; and the sources of information about antibiotics. The intention to SMA and the actual SMA were directly questioned using a dichotomized scale (Yes/No). Socio-demographic characteristics involved gender, age, marital status, household's income per month, highest education achievement, current employment, medical insurance membership and number of family member/s. The questionnaire was assessed for face and content validity by a group of local experts, i.e. three pharmacists, three medical doctors and a psychologist. This consultation process led to redrafting and re-organizing items in the questionnaire. The questionnaire was pilot tested with five people, who were representative of the study population, to determine the clarity of the language used and questionnaire structure. Some words were changed based on responses.

Ethics approval was provided by the Human Research Ethics Committees at The University of Adelaide (H-145-2009; RM No: 9508). Ethical clearance was not needed from the ethics committee in Yogyakarta. Data collection was conducted over 3 months during March to May, 2010 by the five trained survey staff and the first author. The head of the neighborhood was approached to accompany the data collectors in knocking on doors in each neighborhood. If the data collector failed to meet the potential participant in the first visit to the household, a message requesting a further meeting was left for him/her. Three documents (in the Indonesian language) were provided to the selected individuals: a letter describing the aims of the project; a copy of the research permit issued by the Government of Yogyakarta City Indonesia (Pemerintah Kota Yogyakarta Dinas Perizinan: 070/1970/5328/34); and a consent form. The pre-tested questionnaire was self-administered to those who consented to participate in this study. For the illiterate participant, the data collector read the questions in the questionnaire as well as wrote the answers on behalf of the participant. Given local cultural expectations, a small gift was given to the participant on completion of the questionnaire.

### Variables and data analysis

Data were entered, analyzed and digitally stored with the assistance of Statistical Package for the Social Sciences (SPSS) version 17. Descriptive statistics were used to describe socio-demographic characteristics of the respondents, the point prevalence and the patterns of self medication with antibiotics.

Chi-square test and logistic regression were applied. The predictor variables were the socio-demographic characteristics, which included age, gender, marital status, highest education achievement, household's income level, health insurance, and number of family members living in the same house. The criterion variables were the intent and the actual SMA.

## Results

From 640 selected households, 625 potential respondents were approached, which excluded fifteen heads of households who could not be reached because they and their family members were no longer living in the selected neighborhoods. Of these 625 potential respondents, 51 refused to participate and 15 withdrew from this study due to being too busy to complete the questionnaire. Finally, 559 respondents completed the questionnaires, which represented a response rate of 90%. Socio-demographic characteristics of respondents are provided in Table 2.

**Table 2:** Demographic and socio-economic characteristics of respondents to self medication with antibiotics survey in Yogyakarta City Indonesia

Demographic and socio-economic characteristics	Number (percentage) of respondents N: 559		
	
	Non- antibiotic users; n = 478	Prescribed antibiotics users; n = 40	Non-prescribed antibiotics users (self medication); n = 41
1. Gender:			
Female	259 (46)	26 (5)	24 (4)
Male	219 (39)	14 (3)	17 (3)
2. Age in years:			
Less than 24	49 (9)	4 (1)	3 (0.5)
24 to 54	293 (52)	31 (6)	34 (6)
54 to 64	78 (14)	4 (1)	2 (0.3)
More than 64	58 (10)	1 (0.1)	2 (0.3)
Median (range)	43 (18-88)	40.5 (18 - 69)	43 (18 - 66)
3. Marital status:			
Married	329 (59)	31 (6)	26 (5)
Unmarried/single	105 (19)	8 (1)	10 (1.8)
Widow/widower	44 (8)	1 (0.1)	5 (1)
4. Household's income per month:			
Less than US $ 150	222 (40)	22 (4)	19 (3)
US $ 150 to US $ 300	155 (28)	9 (1.6)	15 (2.7)
US $ 300 to US $ 800	51 (9)	4 (0.7)	1 (0.1)
More than US $ 800	7 (1.3)	2 (0.3)	2 (0.3)
Did not mention	43 (8)	3 (0.5)	4 (0.7)
5. Highest education achievement:			
University	141 (25)	13 (2)	8 (1.4)
Senior high school	176 (31)	14 (2.5)	17 (3)
Junior high school	59 (11)	5 (0.9)	3 (0.5)
Elementary school	42 (8)	3 (0.5)	5 (0.9)
Did not mention	60 (11)	5 (0.9)	8 (1.4)
6. Current employment/status:			
Unemployed	133 (24)	15 (2.7)	14 (2.5)
Employed	223 (40)	15 (2.7)	19 (3.4)
Did not mention	122 (22)	10 (1.8)	8 (1.4)
7. Medical insurance holder:			
Yes	228 (41)	14 (2.5)	21 (4)
No	240 (43)	23 (4)	17 (3)
Did not mention	10 (2)	3 (0.5)	3 (0.5)

Note: US $ 1 ~ Rp. 10,000 (Indonesian currency); unemployed, i.e.: housewife, retirement, student, jobless; employed, i.e.: private employee, civil servant, entrepreneur, labor.

### A one-month period prevalence of self medication with antibiotics

Intent to self medicate with antibiotics was declared by 324 of 559 respondents (58%). Of the 559 respondents, 40 (7%) had used prescribed antibiotics; 34 (6%) had self medicated with antibiotics; and 7 (1.3%) had used both prescribed and non-prescribed antibiotics during the past month. The 4-week period prevalence of self medication with antibiotics was 7.3%.

### The patterns of use

Although some respondents self medicated with antibiotics for more than once during the past month, only the first period of use was analysed to describe the following patterns of use. Several types of antibiotics, such as amoxicillin, ampicillin, ciprofloxacin, and tetracycline, were used to treat a variety of symptoms including the common-cold, cough, sore throat, headache, ithcing, tootache, fever, etc. Such antibiotics were mainly used for only one to two days. Pharmacies were the most popular retailer/outlet for purchased antibiotics without prescription (64%) besides other un-official vendors. Sources of information about antibiotics purchased were health professionals, friends/relatives, and printed materials. Among the 41 respondents who self medicated with antibiotics, the majority (80%) had previous experience in using such antibiotics; in which such a previous successful experience was the main reason of such use (54%) (Table 3).

**Table 3:** Patterns of self medication with antibiotics among people in Yogyakarta City Indonesia

Patterns of self medication with antibiotics	Number (n: 41)
1. What were the names of the antibiotics you used in the last month?	
Amoxicillin	32
Ampicillin	4
Others (e.g.: fradiomycin-gramicidin, ciprofloxacin, tetracycline)	5

2. What were the health problems/symptoms that you used these antibiotics to treat?	
Common-cold, cough, sore throat	13
Headache	8
Itching	5
Toothache	5
Fever	5
Others (stomach pain, ear pain, post partum)	5

3. Had you used these antibiotics previously?	
Yes	33
No	8

4. Where did you obtain these antibiotics?	
Purchased the antibiotics without prescriptions	29
Combination of purchased them without prescriptions and leftovers	5
Combination of purchased them without prescriptions, leftovers, and obtaining from family or friends.	7

5. If you purchased the antibiotics without prescription, where did you purchase them?	
Pharmacies	26
Combination of pharmacies, drug stores, and kiosks	8
Kiosks	5
Drug stores	2

6. How did you know about these antibiotics? (sources of information about antibiotics)	
Physician, pharmacist	21
Friends, Relatives	13
Combination of pharmacist, pharmacy assistant, drug store owners, and health professionals at primary health care	4
Magazine, advertising, brochure	2
Past experiences	1

7. What was the cost of these antibiotics?	
Less than $ 0.5	12
Free (obtained from relatives/friends; leftovers)	11
$ 0.5 to $ 1	9
$ 1 to $ 3	8
More than $ 3 *Note: US $ 1 ~ Rp. 10,000 (Indonesian currency)*	1

8. How many days did you use this antibiotics?	
One day to two days, if necessary	15
Three to seven days	20
More than seven days	6

9. What were your reasons to use these antibiotics to treat your health problems/symptoms with the non-prescribed antibiotics?	
Previous successful experiences	24
Saving time	5
Saving money	5
Others (easier, doctors tend to prescribe the same antibiotic, recommended by health professionals)	7

### Socio-demographic factors

There were no significant associations between socio-demographic variables and the practice of self medication with antibiotics; N = 559, p > 0.05. On the other hand, gender, marital status, and health insurance were significantly associated with intent to self medicate with antibiotics (N = 559, p < 0.05). Following logistic regression, being male and having no health insurance were 1.7 and 1.5 times more likely be associated with intent to self medicate (Table 4**)**.

**Table 4:** Logistic regression determining likelihood of intents of self medication with antibiotics among people in Yogyakarta City Indonesia

Socio-demographic determinants N = 559	p	Odds ratio	95.0% C.I.
Gender (Male)	0.007	1.7	1.2 - 2.6

Having health insurance (No)	0.036	1.5	1.0 - 2.3

Age (More than 44 years)	0.907	1.0	0.6 - 1.4

Marital status (Married)	0.066	0.6	0.4 - 1.0

Number of family members (More than four people)	0.344	1.2	0.8 - 1.8

Highest education achievement (Senior high school and more)	0.404	0.8	0.5 - 1.3

Household's income (More than US $ 300 per month)	0.425	0.8	0.4 - 1.4

## Discussion

This population-based survey examined randomly selected adults applying a rigorous sampling procedure. The 90% of response rate indicates that the sample is reasonably representative of adult people in Yogyakarta City. The involvement of the head of neighborhoods in knocking on doors, as expected, improved the response rate.

This study identified a 4-week period prevalence of 7.3% for using non-prescribed antibiotics and highlighted patterns of use where amoxicillin, ampicillin, fradiomicyn-gramicidin, ciprofloxacin and tetracycline were the self-medicated antibiotics and were purchased mainly in pharmacies. These antibiotics were used to treat a variety of minor symptoms, such as the common-cold, cough, sore throat, and fever, for mostly less than five days of use. Such practices were based on reasons of previous successful experience, saving time and money, and information obtained from health professionals, lay people, and printed materials.

We acknowledge some limitations in this study. Although this study uses the population of Yogyakarta as its sampling frame, generalization to the whole population of Indonesia should be done carefully. The area of this study, Yogyakarta City, is an urban with a high population density and a mostly literate population. Thus, results may be readily generalizable to other urban areas of Indonesia. For example, the pattern of SMA found in two big cities in Java Island is similar with those in this current study [5]. The situation may well differ in rural areas, however. Further, the use of antibiotics in this study is self reported, and therefore there are some issues regarding subjectivity and imprecision. However, such issues were minimized by using a four-week recall period, which has been extensively used by other similar studies [5,6]. The sample size calculation was based on an estimated period prevalence of self medication with antibiotics of 50%, the midpoint of estimation in the literature. This approach had short comings, however, as the figure is very dependent on the length of the recall period. Unfortunately, our calculation did not account for the variation in these periods. Moreover, because of limited resources a larger precision (d = 0.05) was used to calculate the sample size and a probability proportionate to size (30% and 5% of sub-districts and neighborhoods, respectively) was applied to limit the numbers of clusters in the second and third stages. However, the involvement of all clusters (districts) of the first stage could minimize sample bias [23]. In addition, a quarter of the respondents did not mention their current employment thus this question may be sensitive in the local socio-cultural context. People would perhaps be unwilling to be honest when they are unemployed or non-economically active or employees of informal/casual sectors. Despite such limitations, as a consequence of representativeness of the urban population following the inclusion of a random sample, information yielded from this study is invaluable in describing the use of non-prescribed antibiotics.

A one-month period prevalence of self medication with antibiotics in this study is comparable to that of a household survey in Jordanian population (a one-month period prevalence 9%) [6]. Higher period prevalence has been reported in other developing regions, for example, Mexico (2-week period prevalence 18%) [2] and Sudan (4-week period prevalence 48%) [3]. A four- week period prevalence of 7.3% found in this present study is higher than the previous Indonesian study (a 4-week period prevalence of 3%) [5]. This finding indicates a potential increase in self medication with antibiotics among Indonesians, particularly in urban areas. Further, a four-week period prevalence of 7.3% indicates that an even larger proportion of the urban population in Indonesia would self medicate with antibiotics annually.

Generally, the patterns and the reasons of using non-prescribed antibiotics found in this study are similar to those in other less developed countries [1,2,6,26] and in some European countries [27]. Such patterns, for example, the use of non-prescribed antibiotics for the common-cold, indicate inappropriate use. They also indicate overprescribing of antibiotics as people do tend to duplicate their previous prescriptions for self medication [28,29]. Although in Indonesia and elsewhere prescription scripts are retained in pharmacies, people commonly make notes of prescribed medicines for future reference if similar medical problems reoccur [28,29]. In Indonesia there is a promising intervention program to improve rational prescription of antibiotics particularly for URTI (Upper Respiratory Tracts Infection) known as Monitoring, Training and Planning (MTP) conducted by the Centre for Clinical Pharmacology and Drug Policy Studies. The program has been implemented among health professionals in hospitals in Yogyakarta and has been successfully decreased antibiotics prescription for URTI. Such programs should be applied extensively with the involvement of health professionals in both public and private health service; and private practices.

The patterns of use also indicate inadequate information given to patient as well as to the public. In most developing countries, drug information given by health providers in both primary health care centers and hospitals is not yet optimal [30]. In addition, pharmacy personnel tend to be businessmen rather than professional. When antibiotics are requested by consumers requests are neither refused nor questioned [16]. Since antibiotics are legally grouped as prescription-only medicines, information about such medicines is, unfortunately, not readily available to the general public, but only to health practitioners. It is different with the over-the-counter (OTC) medicines or general sales, where information about the product can be easily accessed through the packed inserts and mass media advertisements. Currently, there is an initiative to locate Indonesian pharmacists in primary health care centers. Such an initiative is promising, particularly to provide adequate information related to medicines including antibiotics and to improve the quality of health care service in general.

Economic considerations are an important factor influencing SMA behavior [29]. There is mixed evidence on the association between lower income and SMA behavior [1,11-15,31]. In this study household income level is not associated with either intent or actual SMA. However, this issue is significant for further investigation in Indonesia because a substantial proportion of the whole Indonesian population is poor [21]. Further, as found in this study, having no health insurance is a significant factor associated with self medicating with antibiotics, which is consistent with a previous Indonesian study [5]. Unfortunately, only approximately 30% of the whole population is covered by health insurance [31].

## Conclusions

Self medication with antibiotics is common among urban population of Indonesia, in which being male and having no health insurance were highlighted to have intent of such use. Underlying factors of why people self medicate with antibiotics should be further examined to better understand this behavior. Given that past experience in using antibiotics is the main reason of the actual SMA, people should be informed that previous antibiotics used are not always appropriate to be used for future medical problems. Impact of health insurance coverage on such behavior should also be further investigated.

## Competing interests

The authors declare that they have no competing interests.

## Authors' contributions

All authors contributed to conception and design of the study. AW carried out data collection, data analysis, data interpretation and drafted the manuscript. JEH, CdeC, and SS checked and clarified data analysis and data interpretation; and revised draft of the manuscript critically. All authors read and approved the final manuscript.
